# Children's television viewing and objectively measured physical activity: associations with family circumstance

**DOI:** 10.1186/1479-5868-3-36

**Published:** 2006-10-25

**Authors:** Kylie Hesketh, David Crawford, Jo Salmon

**Affiliations:** 1 Centre for Physical Activity and Nutrition Research, School of Exercise and Nutrition Sciences, Deakin University, Melbourne, Australia

## Abstract

**Background:**

The contribution of family circumstance to physical activity and television viewing has not been widely investigated in pre-adolescents, and available information is inconsistent. This study examines whether television viewing and objectively measured physical activity vary by different indicators of family circumstance.

**Methods:**

Data from the 2001 Children's Leisure Activity Study and the 2002/3 Health, Eating and Play Study, involving Australian children in Grades Prep (mean age 6y) and 5–6 (mean age 11y), were combined. Children wore accelerometers for six consecutive 24 hour periods. Average min/day in low-intensity activity (1.0–1.9 METs) and moderate-to-vigorous-intensity activity (≥3 METs) were calculated. Parents reported children's television viewing and family circumstance. Linear regression analyses were conducted separately for young girls, young boys, older girls and older boys.

**Results:**

Complete data were available for 2458 children. Parental education and, to a lesser extent, employment level were inversely associated with television viewing. Children in single-parent families, those whose fathers were not in paid employment, and those without siblings tended to spend more time in low-intensity activity than their peers. Children with siblings spent more time in moderate-to-vigorous-intensity activity; associations were stronger for girls. Maternal education was positively associated with moderate-to-vigorous-intensity activity for younger children. Maternal employment was positively associated with moderate-to-vigorous-intensity activity for older children. Multivariable models did not demonstrate a cumulative explanatory effect.

**Conclusion:**

Individual measures of family circumstance were differentially associated with television, low-intensity activity and moderate-to-vigorous-intensity activity and associations were often not consistent across age-by-gender groups. Interventions may need to be tailored accordingly.

## Background

Regular physical activity is associated with improved physical and psychosocial well-being in children [1,2], while frequent television viewing appears detrimental [3]. Consequently, guidelines have been developed outlining recommended minimum physical activity and maximum electronic media use to maintain health [1,4,5]. Many children do not meet these recommendations [6,7], but they are not equally distributed across social and demographic groups. Girls [6,8,9] and older children [6,8,9] are less active. Age and gender are less consistently related to television viewing [10] although some studies of pre-adolescents report girls [7,10,11] and younger children [7,10-12] watch less television.

The family is a potentially important source of influence on children's physical activity and television viewing [13]. However the role of family circumstance, used here to describe family characteristics including individual-level socioeconomic status (SES) and family composition, has been poorly investigated. It is important to consider different indicators of family circumstance as differential associations may point to different underlying pathways. For example, the pathways may relate to differences in parental time, parental knowledge, or financial resources that are available to facilitate children's physical activity involvement.

There is inconsistent evidence for an association between SES, the most commonly investigated aspect of family circumstance, and the physical activity of preadolescent children. A 1999 review [14] of studies considering SES and physical activity associations in 4–12 year olds found positive, negative and no associations were reported. On the balance of evidence the review concluded there was no association. Recent studies also report positive [15-19], negative [20,21], and no associations [15,21-23]. Even within studies findings differ for girls and boys [15,21], different indicators of SES [15] and different measures of physical activity [23]. In contrast, a 2004 review [10] of correlates of television viewing in 2–18 year olds found SES, measured by parent education or income, was consistently inversely related to television viewing. Maternal employment was positively associated with television viewing in some studies and negatively associated in others [10]. However, other measures of individual-level SES (type of school attended and father's occupation) were not associated with children's television viewing [10].

Other indicators of family circumstance have been less widely investigated and have produced conflicting results for physical activity but more consistent results for television viewing. Multiple studies report no association between number of parents in the home and children's physical activity [15,18,21,24]. However, one study reported children in single-parent families are more active [19], and another reported the same finding for boys but not girls [25]. In contrast, children in dual-parent families [16,18] and those with married mothers [17] are typically more physically activity and less sedentary. Television viewing is consistently inversely related to number of parents in the home [10], but generally not associated with the presence of siblings [10]. The only study that measured number of people in the household found no significant association with children's physical activity [15]. One study measured physical activity objectively [23]. No significant associations between vigorous-intensity physical activity and number of parents in the home, parent education or number of children were reported for either gender.

Available information on associations between children's physical activity and family circumstance in the pre-adolescent age group is inconsistent. Most studies have considered single indicators of family circumstance, making it difficult to compare different aspects of family circumstance across studies with different samples and methodologies. Current evidence is further limited by subjective measurement of physical activity. This study aims to examine whether television viewing and objectively measured physical activity vary by different indicators of family circumstance in pre-adolescent children.

## Methods

### Sample

Data were combined from two cross-sectional studies, both assessing family circumstance, television viewing and objectively-measured physical activity in independent samples: 2001 Children's Leisure Activities Study (Study 1) and 2002/2003 Health, Eating and Play Study (Study 2). Both used stratified random sampling with probability proportional to size (total enrolment) to select schools in Melbourne, Australia with more than 200 students. Study 1 selected 19 government schools in high (n = 10) and low (n = 9) SES areas. Study 2 classified government and Catholic schools into SES quintiles using Australian Bureau of Statistics Socio-Economic Indexes for Areas Index of Relative Socio-Economic Disadvantage [26]. Thirteen schools from each included quintile (1^st^, 3^rd ^and 5^th ^quintile; 2^nd ^and 4^th ^quintile schools were excluded) were selected to provide a cross-section of SES; a total of 24 (9 high, 7 middle and 8 low SES) schools agreed to participate.

Due to constraints on the number of schools able to be included in the study, it was not possible to sample schools from all SES quintiles, however the SES of individual families included is likely to have spanned all SES quintiles. For both studies, all children in Grade Prep (mean age 6y) were invited to participate. All Grade 5–6 children (mean age 11y) attending Study 1 schools and 15 of the 24 Study 2 schools were invited to participate (study 2 was initially a study of Grade Prep children only; opportunistically, Grades 5 and 6 children were included during 2003 data collection). The 15 schools from which Grade 5 and 6 children were included were spread across the low, middle and high SES groups.

Studies received ethics approval from the University Ethics Committee and appropriate education authorities. Under existing ethical guidelines it was necessary to seek active consent from parents for each child's participation and no information could be accessed regarding characteristics of non-respondents.

### Measures

Children wore the Manufacturing Technology Inc accelerometer Model 7164 (formerly CSA) on their right hip for six consecutive 24-hour periods except when bathing, swimming and sleeping. It measures intensity, frequency and duration of movement, and is a valid predictor of children's heart rate and energy expenditure [27]. Accelerometers were calibrated and pre-programmed to commence recording in one-minute epochs. Data days were excluded if total recorded movement counts were <10,000 or >20,000,000 within the 24 hour period [28], or if vigorous-intensity activity exceeded six hours. Subjects with fewer than four days of complete data (the threshold for estimating children's habitual physical activity using accelerometers [29]) were excluded.

Movement count thresholds, based on age-specific energy expenditure prediction equations [30], were applied with a QBASIC data reduction program to calculate time (min/day) spent in each intensity of physical activity [31]. Intensities were defined in metabolic equivalents (METs) as sedentary time or low-intensity physical activity (lowPA) = 1.0–1.9 METs and moderate-to-vigorous-intensity physical activity (MVPA) = 3.0 METs. Light-intensity physical activity (2.0–2.9 METs) data were not analysed. Daily time in each intensity was summed and divided by the total number of data days to derive average min/day in lowPA and MVPA. Calculation of lowPA time also involved subtracting usual sleep time (parent-reported) from total daily lowPA time.

Parents reported children's usual television viewing time (including videos and DVDs) via self-completed questionnaire. In Study 1 parents reported total hours and minutes their child spends watching television on Monday-Friday and Saturday-Sunday during a typical week. Estimates were summed then divided by seven to generate average viewing time (min/day). In Study 2 parents reported the time their child spends watching television on a usual weekday and usual weekend day. Weekday estimates were multiplied by five and weekend day estimates were multiplied by two, the totals were summed and divided by seven to generate average viewing time (min/day).

Parents also provided family circumstance information including highest level of education and current employment status of the respondent and co-carer, marital status, number of parents in the home and number of children in the family. Parents in Study 2 also reported average daily hours of paid employment for themselves and the co-carer. Based on gender, respondent and co-carer information was converted to maternal and paternal information.

### Analyses

Due to both studies' cluster-based sampling, analyses included school as the cluster unit (i.e. using *survey *commands with *svyset, psu(school) *and *cluster(school) *options in Stata). As children's physical activity differs by age and gender,[14] analyses were conducted separately for each gender-by-age group combination (young girls, young boys, older girls, older boys). Associations between television, lowPA and MVPA were estimated by Pearson's correlation coefficients (i.e. *corr *command in Stata). Regression analyses compared average time (minutes per day) viewing television, in lowPA an MVPA by gender and age group (e.g., *svyregress lowPA gender *in Stata). For all family circumstance variables, average television, lowPA and MVPA time were calculated. Independent samples t-test analyses (e.g., *ttest lowPA, by(study) *in Stata) revealed differences in television, lowPA and MVPA time by study group (Study 1 versus Study 2; p < 0.001 for all). Therefore, all regression analyses also included study group as a covariate. Linear regression, with linear trend for ordinal predictor variables, estimated levels of association between individual family circumstance variables and each outcome (e.g., *xi: regress lowPA i.maternal_edu study, cluster(school) *and *regress lowPA maternal_edu study, cluster(school) *in Stata). Within regression analyses the Wald test was used to test the joint null hypothesis for multi-category predictor variables using the Stata *testparm *command [32], producing a single p-value for multi-category predictor variables. Multivariable linear regression analyses were conducted to consider the relative contribution of each family circumstance variable to television viewing, lowPA and MVPA time.

## Results

### Sample

Active consent was received for 2772 children; 1210 (38% response) in Study 1 and 1562 (42% response) in Study 2. No area-level socio-economic gradient was noted in response rates for Study 2 (41% response at high, 39% at middle and 48% at low SES area schools) but higher response rates were recorded at high SES schools in Study 1 (53% response at high and 38% response at low SES area school). Due to incomplete data 314 children were excluded. The sample of 2458 reported here contains a similar proportion of girls and boys and more older than younger children; 394 young girls, 386 young boys, 914 older girls and 764 older boys. Most families reported usually speaking English at home (90%), were dual-parent households (84%) and contained multiple children (89%). Families spanned the socioeconomic spectrum: 13% low (<25^th ^percentile), 36% mid (26^th^–75^th ^percentile) and 51% high (>75^th ^percentile) SES (postcode derived Socio-Economic Indexes for Areas Index of Advantage-Disadvantage[33]). Parents were evenly divided by education level; 26% and 23% of mothers and fathers respectively had not completed secondary school, 38% and 44% were secondary educated, and 36% and 33% were tertiary educated.

### Television viewing and physical activity

Objective measurement showed children spent, on average, 369 min/day (95% CI = 365, 374) or just over six hours in sedentary pursuits (lowPA) during their awake time. According to parent-report, approximately half this time can be accounted for by television viewing (mean = 163 min/day; 95% CI = 159, 166). In comparison, children spent only 166 min/day (95% CI = 163, 169) or 2 3/4 hours in MVPA. There was a weak positive association between parent-reported television time and objectively measured lowPA (r = 0.17) and a weak inverse association between television time and MVPA (r = -0.14). LowPA and MVPA were inversely related (r = -0.62).

Older children spent more time watching television (169 versus 148 min/day; p = 0.004) and in lowPA (390 versus 325 min/day; p < 0.001) than younger children, and less time in MVPA (128 versus 245 min/day; p < 0.001). No gender difference was apparent for television (p = 0.44) or lowPA time (p = 0.73). However, girls spent less time than boys in MVPA (154 versus 179 min/day; p < 0.001).

### Relationship between family circumstance and television and physical activity

#### Television viewing and low-intensity time

The strongest predictors of children's television time were maternal and paternal education (Table 1). In all age-by-gender groups, higher parental education was associated with less television viewing. This was not the case for objectively measured sedentary time (lowPA) (Table 2). An inverse association between maternal education and lowPA was observed only in young boys (linear trend p = 0.01). While lowPA was inversely associated with maternal education for older boys (p = 0.01) and with paternal education for older girls (p = 0.03), these relationships were not linear. The strongest predictor of lowPA for younger and older girls was the presence of siblings. Children without siblings spent more time in lowPA than children with siblings. In contrast, television time was similar for children with and without siblings. Television and lowPA time were generally higher for children in single-parent families; differences were stronger for older children. A similar pattern emerged for marital status. Mean television time was lowest for children whose parents were married/de facto. This was significant only for younger and older girls.

**Table 1:** Relationship between family circumstance and minutes per day engaged in television viewing

**Family circumstance**	**young girls**				**young boys**				**older girls**				**older boys**			
**variable**	**n**	**mean**	**β**	**p-value**	**n**	**mean**	**β**	**p-value**	**n**	**mean**	**β**	**p-value**	**n**	**mean**	**β**	**p-value**

Parents in the home				0.14				0.54				0.002				0.13
One^a^	57	158.4	--		52	151.6	--		148	182.9	--		113	176.5	--	
Two	332	145.2	-12.2		323	149.0	-4.3		726	164.4	-21.7		623	170.4	-15.6	
Respondent's marital status				0.01^b^				0.68^b^				0.001 ^b^				0.15^b^
Married/defacto^a^	331	143.9	--		321	148.2	--		715	163.6	--		613	170.0	--	
Separated/divorced	33	150.3	8.6		37	156.1	7.2		123	182.7	22.9		91	170.1	10.5	
Widowed/never married	23	190.1	47.5		15	155.7	11.1		28	189.7	28.2		25	203.2	37.1	
Siblings				0.74				0.79				0.06				0.93
None^a^	40	142.8	--		41	154.1	--		91	179.2	--		75	164.4	--	
One or more	347	147.7	3.6		333	149.1	-2.2		782	166.1	-14.8		657	172.0	1.1	
Maternal education				0.002 ^b ^(0.001)				<0.001 ^b ^(<0.001)				<0.001 ^b ^(<0.001)				<0.001 ^b ^(<0.001)
Not completed secondary^a^	81	159.5	--		85	178.2	--		242	189.0	--		184	193.4	--	
Completed secondary	151	159.4	-2.4		142	155.7	-24.6		306	173.5	-21.3		286	175.8	-19.0	
Tertiary	148	124.7	-34.8		142	125.7	-51.1		293	140.9	-53.1		243	148.8	-46.4	
Paternal education				0.002 ^b ^(0.001)				<0.001 ^b ^(<0.001)				<0.001 ^b ^(<0.001)				0.02 ^b ^(0.04)
Not completed secondary^a^	77	173.3	--		76	173.2	--		166	177.1	--		137	175.5	--	
Completed secondary	144	146.4	-31.3		130	150.5	-25.7		327	173.1	-15.4		279	178.1	3.1	
Tertiary	111	125.1	-51.2		114	127.8	-46.4		238	145.6	-37.8		206	156.1	-19.7	
Maternal employment				0.02 ^b ^(0.55)				0.06 ^b ^(0.03)				0.07 ^b ^(0.09)				0.04 ^b ^(0.02)
Full-time	72	161.9	2.1		67	150.6	-14.8		253	168.6	-18.7		223	165.5	-20.3	
Part-time	148	130.9	-28.6		132	135.2	-26.5		363	159.2	-23.8		279	173.2	-9.6	
Not in paid employment^a^	153	156.2	--		158	162.1	--		205	180.0	--		200	175.5	--	
Paternal employment				0.05 ^b ^(0.03)				0.53 ^b ^(0.91)				0.23 ^b ^(0.17)				0.63 ^b ^(0.34)
Full-time	283	141.8	-72.9		281	148.1	-8.0		625	163.7	-17.3		534	170.1	-13.0	
Part-time	15	176.4	-31.8		17	144.2	-25.4		40	184.8	4.5		32	179.3	-7.2	
Not in paid employment^a^	16	221.7	--		12	152.1	--		35	175.4	--		32	184.6	--	
Maternal work hours (/day)	253		-0.36	0.78	243		-1.86	0.17	398		-2.19	0.14	331		-0.50	0.74
Paternal work hours (/day)	212		-1.64	0.46	204		3.15	0.04	353		-6.32	0.002	303		-0.54	0.75

^a ^referent category; ^b ^Wald test within regression analysis, ( ) p-value for linear trend

**Table 2:** Relationship between family circumstance and minutes per day engaged in low-intensity activity

**Family circumstance**	**young girls**				**young boys**				**older girls**				**older boys**			
**variable**	**n**	**mean**	**β**	**p-value**	**n**	**mean**	**β**	**p-value**	**n**	**mean**	**β**	**p-value**	**n**	**mean**	**β**	**p-value**

Parents in the home				0.14				0.92				0.09				0.05
One^a^	56	354.4	--		54	322.6	--		155	402.6	--		114	409.1	--	
Two	331	319.7	-33.2		327	325.6	2.1		751	387.2	-16.5		639	387.0	-25.5	
Respondent's marital status				0.10 ^b^				0.78 ^b^				0.11 ^b^				0.14 ^b^
Married/defacto^a^	331	320.8	--		327	325.3	--		740	386.5	--		629	387.2	--	
Separated/divorced	32	324.8	5.7		37	334.6	8.8		128	404.2	18.9		95	407.7	24.6	
Widowed/never married	23	381.2	61.0		16	305.2	-18.4		29	405.1	19.5		25	384.5	-1.3	
Siblings				0.005				0.09				<0.001				0.53
None^a^	41	373.3	--		42	361.3	--		96	425.5	--		76	395.7	--	
One or more	346	319.0	-54.5		339	320.7	-38.1		809	385.6	-41.0		676	389.7	-8.4	
Maternal education				0.55 ^b ^(0.77)				0.03 ^b ^(0.01)				0.37 ^b ^(0.41)				0.01 ^b ^(0.10)
Not completed secondary^a^	81	318.0	--		85	349.5	--		256	388.3	--		191	406.9	--	
Completed secondary	149	335.3	15.8		143	328.8	-22.9		316	384.5	-5.4		294	379.8	-27.9	
Tertiary	149	318.1	-0.4		148	305.1	-44.2		298	393.8	4.3		247	388.1	-19.9	
Paternal education				0.81 ^b ^(0.65)				0.92 ^b ^(0.79)				0.03 ^b ^(0.08)				0.36 ^b ^(0.57)
Not completed secondary^a^	77	318.3	--		75	326.2	--		176	385.9	--		141	395.8	--	
Completed secondary	144	323.8	2.9		134	328.7	0.7		334	378.5	-10.9		286	381.8	-14.0	
Tertiary	111	314.6	-5.4		115	322.7	-4.1		243	398.2	10.3		211	387.2	-8.9	
Maternal employment				0.07 ^b ^(0.03)				0.01 ^b ^(0.52)				0.54 ^b ^(0.27)				0.58 ^b ^(0.43)
Full-time	71	356.1	39.0		67	347.9	4.9		255	392.7	8.1		225	389.9	-7.6	
Part-time	150	323.7	7.1		136	297.7	-42.6		376	388.5	5.4		288	386.0	-10.3	
Not in paid employment^a^	151	314.3	--		160	340.4	--		218	382.5	--		208	394.5	--	
Paternal employment				0.20 ^b ^(0.08)				0.24 ^b ^(0.11)				0.07 ^b ^(0.05)				0.17 ^b ^(0.07)
Full-time	284	315.6	-46.7		283	321.2	-112.2		644	385.5	-38.3		545	387.5	-24.9	
Part-time	15	339.9	-18.2		19	342.8	-98.7		41	382.2	-41.7		32	405.8	-7.9	
Not in paid employment^a^	15	367.0	--		12	430.4	--		37	421.5	--		34	413.5	--	
Maternal work hours (/day)	250		2.74	0.22	247		-0.24	0.91	408		0.37	0.82	337		2.88	0.17
Paternal work hours (/day)	210		-3.91	0.07	207		0.34	0.91	361		-3.29	0.19	309		-1.51	0.44

^a ^referent category; ^b ^Wald test within regression analysis, ( ) p-value for linear trend

Young girls were the only group for whom there was a linear trend between lowPA and maternal employment status (p = 0.03). Yet, television viewing was generally highest in children whose mothers were not in paid employment (except young girls). Despite an inconsistent pattern of results across employment categories, the association between maternal employment status and children's television time was relatively strong. The association between lowPA and paternal employment was more consistent. Children whose fathers were not in paid employment had the highest lowPA and television viewing times. For Study 2 children, parent work hours were not consistently related to children's television viewing or lowPA. However, there was a strong inverse association between paternal work hours and television viewing for older girls, (p = 0.002).

#### Moderate-to-vigorous-intensity physical activity

Children with siblings spent more time in MVPA (Table 3); this association was strongest for younger (p = 0.04) and older girls (p = 0.06). Children in dual-parent families tended to be more active than those in single parent families, although this trend was not statistically significant. Maternal education was associated with MVPA for younger children; the relationship was positive for young boys but curvilinear for young girls. In the older age groups there was a positive association between maternal employment and MVPA. However, young boys whose mother was employed part-time engaged in more MVPA than those whose mother was employed full-time or not in paid employment. There was no association between MVPA and maternal employment for young girls, or with respondent's marital status, paternal education, paternal employment, maternal or paternal work hours for any group.

**Table 3:** Relationship between family circumstance and minutes per day engaged in moderate-to-vigorous-intensity physical activity

**Family circumstance**	**young girls**				**young boys**				**older girls**				**older boys**			
**variable**	**n**	**mean**	**β**	**p-value**	**n**	**mean**	**β**	**p-value**	**n**	**mean**	**β**	**p-value**	**n**	**mean**	**β**	**p-value**

Parents in the home				0.27				0.44				0.73				0.17
One^a^	59	224.1	--		54	263.7	--		159	119.7	--		121	137.4	--	
Two	335	237.2	12.6		332	254.3	-9.1		755	118.1	-1.4		643	141.3	5.3	
Respondent's marital status				0.60 ^b^				0.52 ^b^				0.44 ^b^				0.56 ^b^
Married/defacto^a^	334	236.8	--		331	253.9	--		745	117.8	--		632	141.2	--	
Separated/divorced	33	230.9	-6.4		37	263.8	10.1		128	122.2	4.3		96	138.5	-4.3	
Widowed/never married	24	222.8	-14.1		16	263.8	9.4		29	115.4	-2.6		25	143.6	1.8	
Siblings				0.04				0.66				0.06				0.20
None^a^	42	216.7	--		42	250.9	--		97	112.1	--		76	136.9	--	
One or more	350	237.6	20.9		343	256.0	4.4		813	119.1	7.1		680	141.2	5.3	
Maternal education				0.07 ^b ^(0.41)				0.14 ^b ^(0.05)				0.88 ^b ^(0.76)				0.46 ^b ^(0.69)
Not completed secondary^a^	83	248.7	--		86	244.7	--		256	117.4	--		192	138.2	--	
Completed secondary	153	226.6	-21.5		145	256.3	12.2		317	119.1	1.8		296	142.8	5.0	
Tertiary	149	236.5	-12.1		149	262.1	17.3		302	118.5	1.2		248	140.1	2.4	
Paternal education				0.23 ^b ^(0.13)				0.84 ^b ^(0.76)				0.13 ^b ^(0.52)				0.50 ^b ^(0.65)
Not completed secondary^a^	77	248.7	--		77	258.4	--		176	116.9	--		141	140.9	--	
Completed secondary	146	235.9	-11.7		136	251.7	-6.2		338	120.8	4.5		290	143.1	2.1	
Tertiary	112	232.1	-15.8		116	254.8	-3.4		243	115.5	-1.1		211	139.3	-1.5	
Maternal employment				0.46 ^b ^(0.29)				0.01 ^b ^(0.06)				0.24 ^b ^(0.09)				0.38 ^b ^(0.18)
Full-time	72	232.5	-7.6		67	254.7	11.2		257	120.9	5.6		225	142.2	5.3	
Part-time	150	229.5	-10.7		137	267.7	23.3		379	118.7	3.3		290	141.9	4.6	
Not in paid employment^a^	156	241.1	--		163	244.5	--		218	115.5	--		210	138.2	--	
Paternal employment				0.90 ^b ^(0.68)				0.42 ^b ^(0.31)				0.82 ^b ^(0.91)				0.94 ^b ^(0.89)
Full-time	286	238.9	2.5		287	255.9	40.2		648	118.2	3.0		549	140.5	1.5	
Part-time	15	233.0	-5.1		20	261.1	47.3		41	122.4	7.2		32	141.4	2.9	
Not in paid employment^a^	16	234.5	--		12	216.6	--		37	115.6	--		34	138.6	--	
Maternal work hours (/day)	255		-0.79	0.47	250		0.36	0.71	411		0.65	0.25	338		-0.29	0.75
Paternal work hours (/day)	212		-0.30	0.82	210		-0.86	0.59	364		1.21	0.13	310		0.65	0.34

^a ^referent category; ^b ^Wald test within regression analysis, ( ) p-value for linear trend

#### Multivariable models

Univariable models accounted for ≤5% of the variance in lowPA and MVPA but up to 19.5% of the variance in television viewing. To assess the cumulative contribution of family circumstance variables, multivariable models including parent education (highest of maternal and paternal education), parent employment (highest of maternal and paternal employment), siblings and number of parents in the home were generated for each outcome (Table 4). To avoid losing subjects the following variables were excluded from multivariable analyses: parental work hours (available for Study 2 participants only); the lower of maternal or paternal education and employment (both variables were not available for children in single-parent families); marital status (which contained similar information to number of parents in the home [Spearman's r = -0.86]).

**Table 4:** Multivariable regression models exploring relationships between family circumstance variables and television viewing, sedentary/low-intensity activity and moderate to vigorous-intensity activity time

**Family circumstance variable**	**Television viewing**		**Low-intensity activity**		**Moderate-to-vigorous-intensity activity**	
	
	**β (95% CI)**	**p-value**	**β (95% CI)**	**p-value**	**β (95% CI)**	**p-value**
**YOUNG GIRLS**		R2 = 0.13		R2 = 0.05		R2 = 0.03
Dual-parent family	-14.19 (-35.64, 7.26)	0.19	-18.12 (-67.01, 30.78)	0.56	5.51 (-17.77, 28.79)	0.64
Siblings	8.40 (-17.58, 34.38)	0.52	-45.55 (-89.55, -1.56)	0.04	18.76 (-0.89, 38.41)	0.06
Parental education	-23.07 (-33.58, -12.56)	<0.001	1.00 (-14.54, 16.54)	0.90	-6.50 (-16.23, 3.25)	0.19
Parental employment	-7.04 (-19.86, 5.78)	0.27	9.94 (-6.76, 26.64)	0.24	-2.65 (-11.25, 5.95)	0.54
**YOUNG BOYS**		R2 = 0.17		R2 = 0.04		R2 = 0.03
Dual-parent family	-3.24 (-16.14, 9.67)	0.62	11.06 (-33.25, 55.36)	0.62	-12.39 (-33.86, 9.09)	0.25
Siblings	-8.47 (-26.13, 9.19)	0.34	-44.46 (-88.93, 0.00)	0.05	9.73 (-8.45, 27.90)	0.29
Parental education	-23.53 (-33.42, -13.64)	<0.001	-13.61 (-28.03, 0.80)	0.06	6.59 (-2.05, 15.22)	0.13
Parental employment	-7.04 (-15.73, 1.65)	0.11	-5.33 (-20.72, 10.06)	0.49	6.96 (-0.97, 14.88)	0.08
**OLDER GIRLS**		R2 = 0.19		R2 = 0.03		R2 = 0.01
Dual-parent family	-18.54 (-32.02, -5.06)	0.009	-13.58 (-34.98, 7.81)	0.21	-2.41 (-12.10, 7.28)	0.62
Siblings	-14.16 (-27.66, -0.67)	0.04	-40.41 (-61.07, -19.75)	<0.001	8.69 (0.57, 16.82)	0.04
Parental education	-26.00 (-33.13, -18.87)	<0.001	3.29 (-3.60, 10.18)	0.34	0.07 (-3.10, 3.23)	0.97
Parental employment	-4.78 (-14.80, 5.25)	0.34	1.03 (-6.79, 8.85)	0.79	2.54 (-0.68, 5.76)	0.12
**OLDER BOYS**		R2 = 0.16		R2 = 0.02		R2 = 0.02
Dual-parent family	-16.14 (-36.43, 4.15)	0.12	-19.44 (-42.93, 4.05)	0.10	4.37 (-3.13, 11.87)	0.24
Siblings	5.63 (-17.77, 29.03)	0.63	-5.91 (-33.56, 21.72)	0.67	4.96 (-3.38, 13.30)	0.24
Parental education	-17.40 (-25.80, -9.00)	<0.001	-9.41 (-21.42, 2.61)	0.12	-1.64 (-6.52, 3.24)	0.50
Parental employment	-8.37 (-16.81, 0.07)	0.05	-0.85 (-12.35, 10.66)	0.88	1.55 (-2.09, 5.19)	0.39

Multivariable models accounted for 13–19%, 2–5% and 1–3% of the variance in television, lowPA, and MVPA time respectively. A greater proportion of variance in objectively measured activity was accounted for by the models for young girls and boys. After accounting for all other family circumstance variables and study group, only parental education significantly predicted children's television viewing for all age-by-gender groups. It was also inversely associated with lowPA in young boys. Presence of siblings was inversely associated with television viewing and lowPA, and positively associated with MVPA for older girls; it was also inversely associated with lowPA for younger girls and boys and positively associated with MVPA for younger girls. Older girls in dual-parent families spent less time watching television than their peers in single-parent families. Parent employment was inversely associated with television viewing in older boys and positively associated with MVPA in young boys.

## Discussion

The aim of this study was to examine whether television viewing and objectively measured physical activity are associated with various indicators of family circumstance in pre-adolescent children. Some of the family circumstance variables were more consistently associated with physical activity, across activity intensities and age-by-gender groups, than others. Generally the measures of family composition (number of parents in the home and presence of siblings) were more consistently related to children's physical activity than were the socioeconomic indicators. Family circumstance appeared to be more strongly related to television viewing (both in strength and number of associations) than to lowPA or MVPA time.

There appeared to be no cumulative explanatory effect of family circumstance in the multivariable models, and vast majority of variance in television viewing, lowPA and MVPA remained unaccounted for. The small amount of variance accounted for by family circumstance (particularly for objectively measured activity) suggests that there are multiple additional factors, not measured in this study, which contribute to or explain children's activity levels. Physical activity is a multidimensional behaviour which is likely to be influenced by a myriad of factors. For other multidimensional issues, such as childhood overweight, it has been determined that the influence of any single factor is weak [34]. This is likely to be the case for physical activity also. Factors not measured in this study including time spent outdoors, neighbourhood characteristics (such as access to programs and facilities), and individual child characteristics (such as activity preferences and intention to be active) [14] are also likely to explain some of the individual-child variance in activity levels. The findings of this study suggest that family circumstance is associated with children's television viewing and levels of physical activity but is not the sole contributing factor.

This study found children in dual-parent families tended to spend less time watching television and engaged in lowPA and more time in MVPA. Similar results were noted for marital status, which may be a proxy for number of parents in the home. However, having a father who was not in paid employment appeared to be a risk factor for high levels of television viewing and lowPA, particularly amongst younger children. It may be that this group of fathers are modelling sedentary behavior. While the vast majority of the sample were reported to have a father in full-time employment, the subgroup with fathers not in paid employment are of interest and may be an important target for intervention.

Children with siblings spent less time in lowPA and more time in MVPA. A review of correlates of physical activity [14] reported higher levels of physical activity among adolescents with siblings but this variable has rarely been studied in pre-adolescents. Children in smaller families may have fewer opportunities for companion play and therefore spend more time in solitary pursuits, many of which are sedentary. Alternatively, parenting practices may differ for single- and multiple-child families; children without siblings may have more restrictions on their outdoor play and/or greater access to sedentary play alternatives.

While television viewing is only one of many activities that could be included in lowPA, it is interesting to note that television viewing was more strongly related to family circumstance than the objective measure of sedentary time (lowPA). It is difficult to know whether this indicates a difference in television viewing behavior per se or a difference between parent-reports and objective measurements. It is possible that there is some relationship between parent-report (of television viewing or any other activity) and family circumstance related to aspects of social desirability and associated reporting bias. Equally, television viewing may be inherently different from other types of sedentary behavior [35]. Unlike television viewing, objectively measured sedentary time comprised a range of sedentary behaviors, many of which may be non-discretionary, occurring outside the home, and thus less likely to be influenced by family circumstance.

These findings are based on cross-sectional data, therefore causality can not be inferred. While the modest response rate might be considered a limitation, participants spanned a broad range of sociodemographic and family circumstance backgrounds. The strengths of the study include the objective measurement of physical activity and a large sample enabling stratification by age and gender. However, it should be noted that where the number of children in a single family circumstance category was low (eg. children with unemployed fathers), estimates should be interpreted with caution.

## Conclusion

Our findings that individual measures of family circumstance were differentially associated with varying intensities of activity and that associations differed across the age-by-gender groups may help explain conflicting findings reported in the literature [14]. This study highlights the complexity of relationships between family circumstance and physical activity and sedentary behavior. Programs promoting physical activity and reducing sedentary time may therefore need to tailor their approach dependent upon the age, gender and family circumstance of the target audience.

## Competing interests

The author(s) declare that they have no competing interests.

## Authors' contributions

KH carried out the statistical analyses and drafted the paper. DC was involved in the design and conduct of both studies and contributed to the drafting of the paper. JS was involved in the design and conduct of both studies and contributed to the drafting of the paper. All authors read and approved the final manuscript.
